# Derivation and Characterization of Endothelial Cells from Porcine Induced Pluripotent Stem Cells

**DOI:** 10.3390/ijms23137029

**Published:** 2022-06-24

**Authors:** Yang Yu, Xuechun Li, Yimei Li, Renyue Wei, Hai Li, Zhonghua Liu, Yu Zhang

**Affiliations:** 1 College of Life Science, Northeast Agricultural University, Harbin 150030, China; y15776623880@163.com (Y.Y.); lixuechun217@neau.edu.cn (X.L.); liyimei2020@163.com (Y.L.); weirenyue217@neau.edu.cn (R.W.); 2 School of Basic Medical Sciences, Xi’an Jiaotong University, Xi’an 710061, China; lihai@xjtu.edu.cn

**Keywords:** pig, endothelial cells, TGF-β, hindlimb ischemia, angiogenesis

## Abstract

Although the study on the regulatory mechanism of endothelial differentiation from the perspective of development provides references for endothelial cell (EC) derivation from pluripotent stem cells, incomplete reprogramming and donor-specific epigenetic memory are still thought to be the obstacles of iPSCs for clinical application. Thus, it is necessary to establish a stable iPSC-EC induction system and investigate the regulatory mechanism of endothelial differentiation. Based on a single-layer culture system, we successfully obtained ECs from porcine iPSCs (piPSCs). In vitro, the derived piPSC-ECs formed microvessel-like structures along 3D gelatin scaffolds. Under pathological conditions, the piPSC-ECs functioned on hindlimb ischemia repair by promoting blood vessel formation. To elucidate the molecular events essential for endothelial differentiation in our model, genome-wide transcriptional profile analysis was conducted, and we found that during piPSC-EC derivation, the synthesis and secretion level of TGF-β as well as the phosphorylation level of Smad2/3 changed dynamically. TGF-β-Smad2/3 signaling activation promoted mesoderm formation and prevented endothelial differentiation. Understanding the regulatory mechanism of iPSC-EC derivation not only paves the way for further optimization, but also provides reference for establishing a cardiovascular drug screening platform and revealing the molecular mechanism of endothelial dysfunction.

## 1. Introduction

Globally, the number of patients with cardiovascular disease (CVD) increases year by year, from 271 million in 1990 to 523 million in 2019 [1]. With the characteristics of poor prognosis and high disability and death rates, cardiovascular disease seriously affects people’s quality of life. Endothelial cells (ECs), located on the inner side of vascular lumen, play critical roles in maintaining vascular integrity, regulating vascular permeability, angiogenesis and vasculogenesis. Loss or dysfunction of ECs is one of the main pathogenic factors of CVD [2,3].

In 1997, Asahara et al., isolated the endothelial progenitor cells from human peripheral blood for the first time and showed that these cells could participate in neovascularization [4]. Subsequent studies suggested that autologous transplantation of CD34^+^ cells improved the perfusion of ischemic tissue and reduced the amputation rate of patients with critical limb ischemia [5]. To mimic human ischemic diseases, animal models of myocardial ischemia and limb ischemia are usually generated to evaluate the effects of cell or drug treatments [6]. However, the limited source and the diseases with endothelial lesions hinder the clinical application of autologous endothelial cells. The emergence of iPS technology theoretically solved the problem; thereafter, an increasing number of studies focused on the directed differentiation of iPSCs into endothelial cells [7,8]. In 2009, a study showed that the morphology and function of ECs derived from human iPSCs were highly consistent with those derived from human ESCs, and both expressed classical endothelial marker CD31, absorbed low-density lipoprotein and formed vascular networks in vitro and in vivo, but compared with ESCs, the differentiation efficiency of iPSCs was lower [9].

During embryonic development, mesoderm segregates from mesendoderm, and a part of mesodermal cells differentiate into endothelial progenitors [10]. The induction of mesendoderm is evolutionarily well-organized and rigorously controlled by several signaling pathways, including Nodal, Wnt and FGF, and a series of transcription factors, including Mix-like, GATA, Sox and Fox [11,12]. Specialized endothelial progenitors will finally become endothelial cells, and this process is regulated by Nodal, BMP4, etc., signaling pathways. Factors such as VEGF and Ihh secreted by primitive endoderm are also involved in endothelial cell formation [13,14,15]. Following the developmental process, researchers tried to derive endothelial cells from pluripotent stem cells in vitro. In 2013, Tan et al., found that short-term treatment of the GSK3β signaling pathway inhibitor CHIR99201 was sufficient for pluripotent stem cells to generate mesendodermal cells [16]. In addition, other small molecules, factors and even microRNAs are proved to promote mesodermal or endothelial generation [17,18,19,20]. Although the study on the molecular mechanism of endothelial differentiation from the perspective of development provides references for endothelial cell derivation from pluripotent stem cells, incomplete reprogramming and donor-specific epigenetic memory are still thought to be the obstacles of iPSCs for clinical application. Thus, it is necessary to establish a stable iPSC-EC induction system and investigate the regulatory mechanism of endothelial differentiation.

Compared with small experimental animals, pigs are considered as ideal cardiovascular and metabolic disease models for humans, due to their similar organ size, anatomical structure and physiological characteristics; moreover, pigs have a relatively long lifespan [21,22,23]. However, compared with humans and mice, studies on porcine vascular endothelial cells are very limited. Although several studies have utilized porcine cardiovascular disease models for cell-based transplantation therapy, most of the transplanted endothelial cells were derived from porcine mesenchymal stem cells [24,25]. In 2013, Gu et al., obtained vascular endothelial cells from porcine iPSCs for the first time by embryoid body differentiation and their subsequent study showed that the cells could improve the cardiac function of mice with myocardial infarction [26]. In 2020, we provided a feeder- and serum-free culture system for deriving ECs from porcine iPSCs, which was the first trial on other species except for human and mice [27,28].

In this study, we examined the in vitro and in vivo angiogenic potentials of porcine iPSC-derived ECs and explored the regulatory mechanism of endothelial differentiation in our culture system. Understanding the mechanism of endothelial differentiation not only paves the way for the optimization of the iPSC-EC induction system, but also provides reference for establishing a cardiovascular drug screening platform and revealing the molecular mechanism of endothelial dysfunction.

## 2. Results

### 2.1. Generation and Characterization of piPSC-ECs

**Figure 1:**
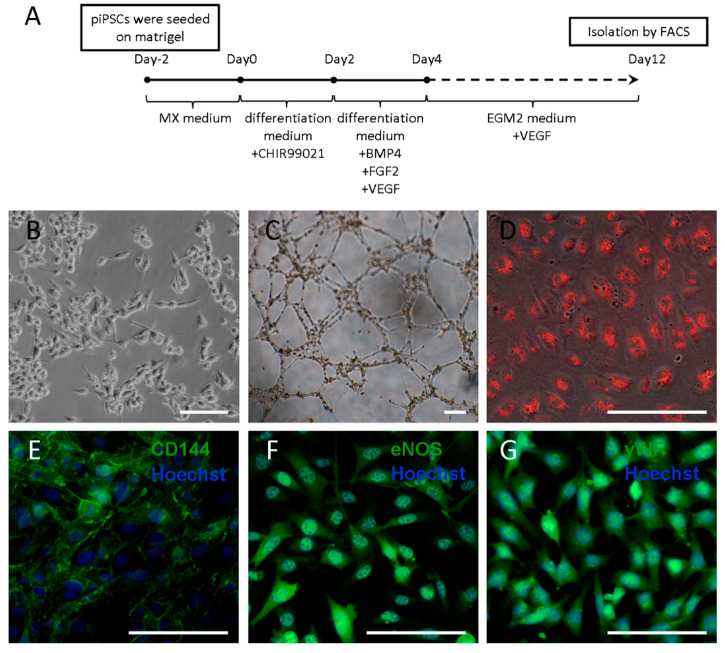
Generation and characterization of piPSC-ECs. (**A**) The schematic diagram for piPSC-EC derivation. (**B**) Morphology of piPSC-ECs. (**C**) Tube-like structures were formed by piPS-ECs. (**D**) Uptake of Dil-Ac-LDL by piPSC-ECs. (**E**–**G**) Endothelial-specific markers were detected via immunofluorescence staining. piPSC-ECs were positive for CD144 (**E**), eNOS (**F**) and vWF (**G**). Blue fluorescence represents the nuclei labeled by Hoechst33342. Scale bar = 50 μm.

The induction protocol of piPSCs into endothelial cells (ECs) is shown in Figure 1A. On day 8 of induction, CD31-positive cells emerged, and on day 12, flow cytometry sorting was used to obtain the CD31-positive cells, which accounted for 10.18% of the differentiated cells (Figure S1). The sorted cells adhered and exhibited typical morphological characteristics of endothelial cells such as diamond or polygon (Figure 1B). In addition to CD31, it also expressed other endothelial markers, such as CD144, endothelial nitric oxide synthase (eNOS) and von Willebrand factor (vWF) (Figure 1E–G). The sorted cells could form tubules in vitro when seeded onto Matrigel and uptake low-density lipoprotein (Figure 1C,D).

### 2.2. In Vitro and In Vivo Angiogenesis of the Derived piPSC-ECs

**Figure 2:**
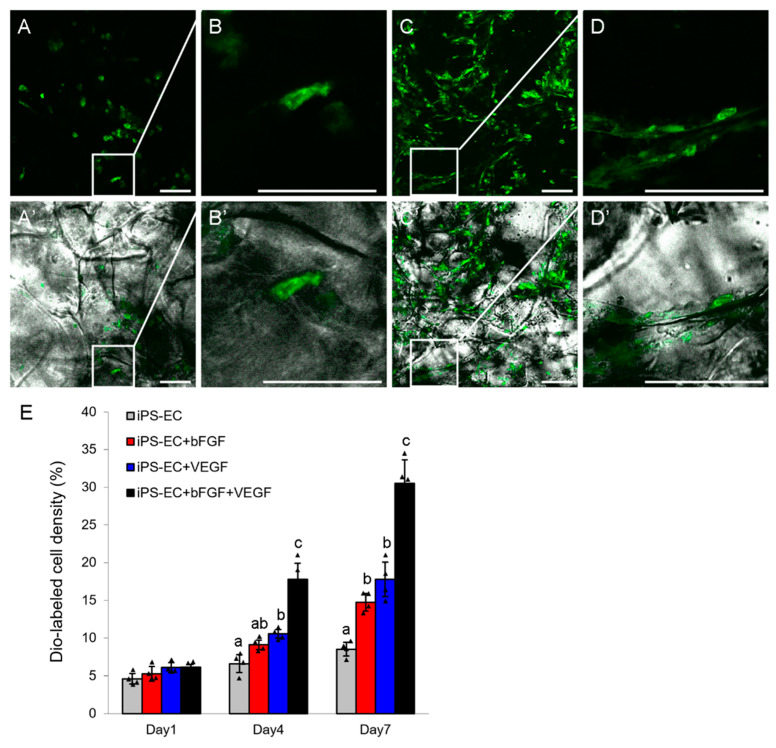
piPSC-ECs cultured on 3D gelfoam scaffolds. (**A**–**D**) Confocal images of Dio-labeled piPSC-ECs on gelfoam scaffolds after 1 day (**A**-**A**’,**B**-**B**′) or 7 days of cultivation (**C**-**C**′,**D**-**D**′). (**B**-**B**′,**D**-**D**′) are the zoomed-in versions of (**A**-**A**′,**C**-**C**′) within the white rectangles. Scale bar = 50 µm. (**E**) Cell density assay was performed at the indicated treatments and time points. Mean ± SD. *n* = 4. The bars without the same letter are significantly different (*p* < 0.05).

piPSC-ECs were seeded onto 3D gelatin scaffolds for in vitro angiogenic assay via confocal microscopy. piPSC-ECs, which were labeled by Dio cell tracker dye, adhered to the gelatin fibers and stretched after one day (Figure 2A,B). After 7 days of cultivation, the number of piPSC-ECs increased and the cells formed tubular structures along the gelatin fibers, establishing a network (Figure 2C,D). Under the stimulation of bFGF or VEGF, the number of piPSC-ECs increased as time went by, and a synergistic effect was observed when the two cytokines were used simultaneously (Figure 2E). These results suggest that piPSC-ECs not only attached and spread on 3D gelfoam scaffolds, but also proliferated under cytokine stimulation as endogenous endothelial cells [29,30,31].

In order to learn the in vivo angiogenesis of derived piPSC-ECs, a hindlimb ischemia model was established by ligating the femoral artery of NOD/SCID mice, and the ligation effect was evaluated by a comparative experiment (Figure S2A–D). The transplanted cells were labeled with PKH26, with the positive rate of 98.74% (Figure S2E,F). A small animal fluorescence imaging system was used for locating the transplanted cells, while the fluorescent signal could only be observed from a few mice on day 7, and on day 14, no fluorescent signal could be observed (Figure S2G–I).

**Figure 3:**
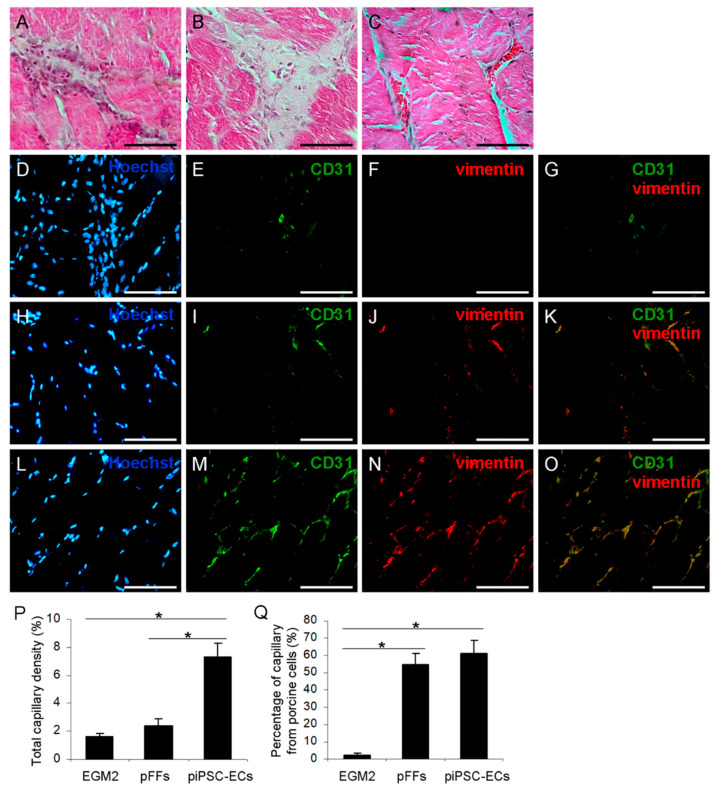
Histomorphology and angiogenesis of adductors after cell transplantation. (**A**–**C**) Histomorphological observation of mice treated with EGM-2 (**A**), PFFs (**B**) and porcine iPS-ECs (**C**). Scale bar = 100 μm. (**D**–**O**) Angiogenesis of mice treated with EGM-2 (**D**–**G**), PFFs (**H**–**K**) and porcine iPSC-ECs (**L**–**O**). Blue fluorescence represents the nuclei labeled by Hoechst33342 (**D**,**H**,**L**). Green fluorescence represents CD31 expression (**E**,**I**,**M**). Red fluorescence represents porcine-specific vimentin expression (**F**,**J**,**N**). Merged images of (**E**,**F**), (**I**,**J**), and (**M**,**N**) (**G**,**K**,**O**). Scale bar = 100 μm. (**P**,**Q**) Quantitative analysis of angiogenesis in different groups. Total capillary density (**P**). Percentage of capillary from porcine cells (**Q**). Mean ± SD, *n* = 6. The bars labeled with an asterisk are significantly different (*p* < 0.05).

Twenty-eight days after ischemic induction and cell transplantation, HE staining was performed for histomorphological observation. The results showed that the adductor muscle from the endothelial cell growth medium-2 (EGM-2)-treated group was partially dissolved and necrotic, accompanied by scar tissue formation and inflammatory cell infiltration; that from the porcine fetal fibroblasts (PFFs)-treated group was relatively complete, but the extensive fibrous structure was visible; the piPSC-EC-treated group had better muscle morphology, and a few microvascular-like structures could be observed (Figure 3A–C). Immunohistochemical results showed that on day 28, a few CD31-positive vascular-like structures appeared in the EGM-2-treated group, and even more CD31-positive vascular-like structures were observed in the PFF- and piPSC-EC-treated groups, some of which were derived from the transplanted cells, which were labeled with porcine-specific anti-vimentin antibody (Figure 3D–O). The results of statistical analysis showed that total capillary density of the piPSC-EC-treated group was 7.32 ± 0.93%, which was significantly higher than those of the PFF-treated group (2.42 ± 0.54%) and the EGM-2-treated group (1.61 ± 0.28%). Among the capillaries, the piPSC-EC-derived capillaries accounted for 61.26 ± 7.33% and the PFF-derived capillaries accounted for 54.70 ± 6.36%, and there was no significant difference between the two groups (Figure 3P,Q).

### 2.3. Genome-Wide Transcriptional Profile Analysis for the Regulation of piPSC-EC Differentiation

To elucidate the molecular mechanism underlying the endothelial differentiation in our model, genome-wide transcriptional profile analysis was conducted. RNA isolated from cells at indicated stages—including porcine iPS cells (IPS), porcine iPS cells after 2 days of endothelial induction (IEC2), porcine iPS cells after 4 days of endothelial induction (IEC4), porcine iPS cells after 12 days of endothelial induction (IEC12) and IEC12 after flow cytometry sorting for CD31-positive cells (S)—was subjected to RNA sequencing, and the reliability of the sequencing data was verified (Figure S3A,B).

**Figure 4:**
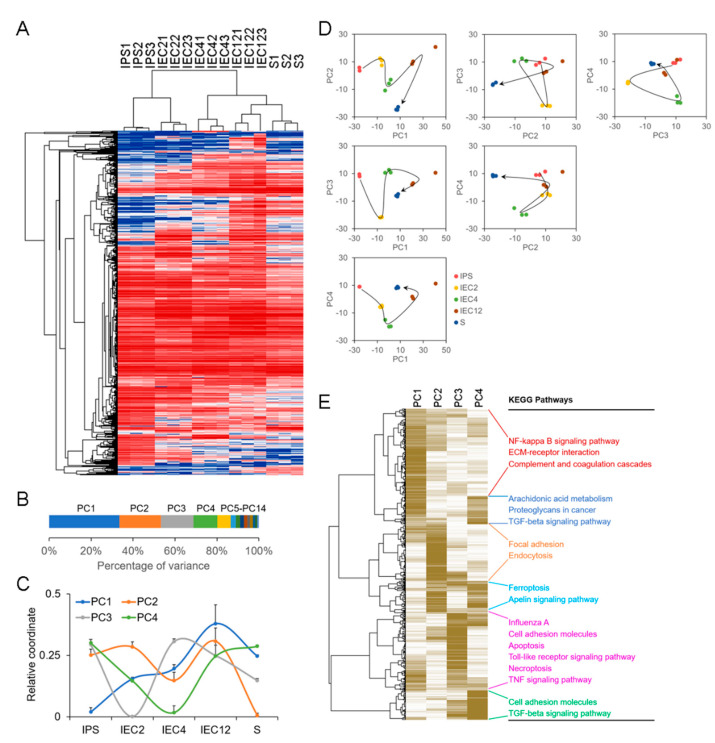
Genome−wide transcriptional profile analysis for the regulation of piPSC−EC differentiation. (**A**) Hierarchical clustering analysis of the genes differentially expressed between iPS cells and AOCs at *p* < 0.001, *q* < 0.001, |log2 fold change| > 3. Columns indicate samples and rows indicate genes. Blue indicates repression and red indicates promotion. (**B**) Principal component analysis (PCA) with the differentially expressed genes using R package ade4 (version 1.7-18). (**C**) The shift in each PC during the process of differentiation. A relative coordinate was calculated by normalizing the coordinates of all cells within each PC with a range from 0 to 1. The results are presented as the mean ± SD, *n* = 3. (**D**) Trajectory visualization in 2D using any two of the top four PCs. (**E**) Heatmap of the high contribution genes of the top four PCs. Columns indicate PCs and rows indicate genes. Brown indicates high contribution and white indicates low contribution. Pathway analysis of indicated genes was performed (*p* < 0.05).

We obtained the molecular events essential for the differentiation of endothelial cells from iPS cells by two steps. First, we compared the transcriptional profiles between iPS cells and AOCs and identified genes differentially expressed between them based on the following criteria: (i) *p* value < 0.001, (ii) q value < 0.001 and (iii)|log2 fold change| > 3, which narrowed down the number of genes essential for endothelial differentiation to 1018 genes (Table S3). Hierarchical clustering analysis using the differentially expressed genes demonstrated efficient clustering of biological replicates for each group. Among these groups, the genome-wide transcriptional profiles of iPS and IEC2 were clustered together, and the genome-wide transcriptional profiles of S and IEC12 were clustered together (Figure 4A). Next, principal component analysis (PCA) using the 1018 genes was conducted using R package ade4 (version 1.7-18) (https://github.com/sdray/ade4 (accessed on 21 October 2021)) to further extract the genes essential for the endothelial differentiation. Fourteen principal components (PCs) were obtained (Figure 4B). The top four PCs contributed more than 80% of the variances among the transcriptional profiles of iPS, IEC2, IEC4, IEC12 and S, and were considered as the main PCs. To monitor the shift in each PC during endothelial differentiation, the relative coordinate was calculated by normalizing the coordinates of the groups within each PC with a range from 0 to 1. As shown in Figure 4C, the coordinate of PC1 started to shift at the beginning of differentiation and basically moved to the same direction throughout the process of differentiation. The coordinate of PC4 also started to shift at the beginning of differentiation but turned back from the stage of IEC4 and reached the original site at the stage of S. The coordinates of PC2 and PC3 changed directions three and two times the number of waves shown in Figure 4C, respectively. To further evaluate the contributions of PCs to the endothelial differentiation in our model, the groups were visually presented in two demotions (2D), and consistent with what we observed in Figure 4C, PC1 and PC4 presented the differentiation trajectory from iPS to S more clearly than other combinations of PCs, suggesting the two PCs as the key components directing the differentiation process (Figure 4D). The genes with high contribution to each PC are clustered in the heatmap in Figure 4E. Functional characterization showed that most of the genes that highly contributed to PC1 were enriched in the NF-kappa B signaling pathway and ECM–receptor interaction, and the rest were shared with those of PC4 and enriched in arachidonic acid metabolism and the TGF-β signaling pathway. Interestingly, the genes that uniquely contributed to PC4 were also enriched in the TGF-β signaling pathway. In fact, the pathways presented in Figure 4E, including the NF-kappa B signaling pathway, ECM–receptor interaction and arachidonic acid metabolism, have been proven to cross-talk with or under the control of the TGF-β signaling pathway [32,33].

### 2.4. Effects of TGF-β on the Differentiation of Porcine iPSC-ECs

**Figure 5:**
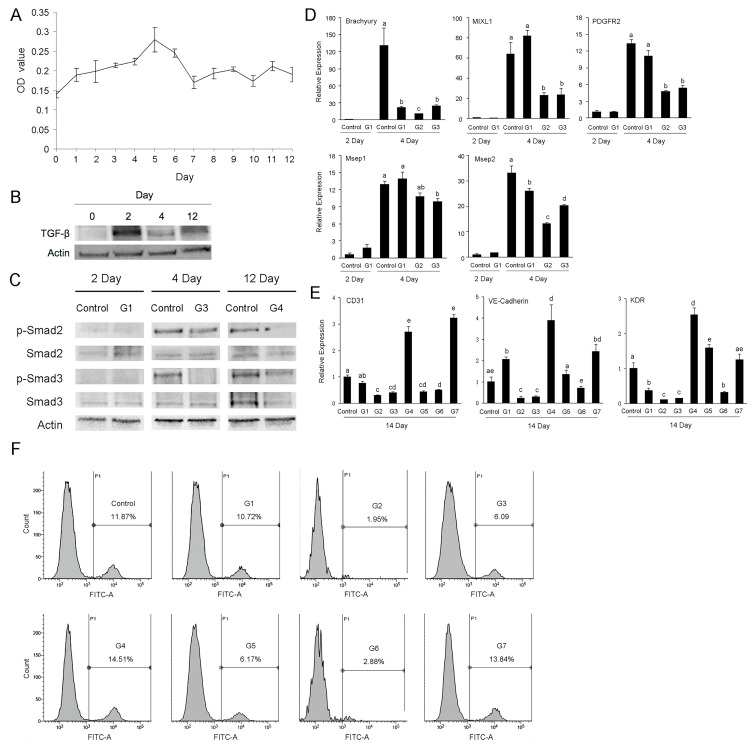
Effects of TGF-β-Smad2/3 signaling on the differentiation of piPSC-ECs. (**A**) Detection of secretion levels of TGF-β1 by ELISA. Mean ± SD. *n* = 4. (**B**) Detection of expression levels of TGF-β by Western blot. (**C**) Detection of Smad2/3 phosphorylation levels by Western blot. (**D**,**E**) Expression levels of mesoderm marker genes (**D**) and endothelial marker genes (**E**) at indicated time points by RT-PCR. Mean ± SD. *n* = 4. The bars without the same letter are significantly different (*p* < 0.05). (**F**) Flow cytometric analysis of CD31-positive cells in different treatment groups on day 12.

Considering the above results from bioinformatic analysis and the roles of TGF-β signaling in the development of human and mouse endothelial cells [34,35,36,37], we firstly detected the secretion level of TGF-β1 during the derivation of porcine iPSCs-ECs by ELISA. The results showed that at the early stage of differentiation, the secretion level of TGF-β1 was low, while along with the prolongation of culture time, the level gradually increased and reached the peak on day 5, and then it decreased and maintained a low level (Figure 5A). The results from Western blot showed that on the second day of differentiation, the expression level of TGF-β1 was highest, followed by a decrease on day 4 and a slight increase on day 12 (Figure 5B). From the above results, we can see that during the differentiation of porcine iPSCs into ECs, both expression and secretion levels of TGF-β1 exhibited dynamic changes.

To know whether and how TGF-β signaling participates in the differentiation of porcine iPSCs into endothelial cells, we set up eight experimental combinations according to the stages of endothelial induction. As shown in Table 1, TGF-β signaling inhibitor SB431542 was added at different time points. SB431542 is a TGF-β type I receptor inhibitor with no effect on BMP signaling [38]. On days 2, 4 and 12 of differentiation, we detected the expression and phosphorylation levels of Smad2 and Smad3 by Western blot, both of which are intracellular transducers of the TGF-β signaling pathway. The results showed that the phosphorylation levels of Smad2 and Smad3 increased on day 4 and remained high until day 12; the phosphorylation levels of Smad2 and Smad3 decreased when SB431542 was added, no matter during the period of day 0–2, day 3–4 or day 5–12 (Figure 5C). The above results indicate that during the endothelial differentiation of porcine iPSCs, TGF-β-Smad2/3 signaling was activated and SB431542 effectively prevented TGF-β signaling transduction.

We collected the cells from differently treated groups on day 2 and day 4 of induction, and detected the expression levels of mesodermal marker genes by RT-qPCR. The results showed that on day 2 of induction, the expression levels of mesodermal markers in the G1 group and the control group were low and there was no significant difference; on day 4 of induction, the early mesoderm marker gene Brachyury was highly expressed in the control group but not in G1, G2 or G3 groups (*p* < 0.05). The expression patterns of late mesodermal marker genes PDGFR2, MXL1, Mesp1 and Mesp2 were similar: their expression levels were higher in control and G1 groups and lower in G2 and G3 groups (*p* < 0.05) (Figure 5D). We examined the expression levels of endothelial marker genes on day 14 of differentiation. The results showed that, compared with the control group, the expression levels of endothelial-specific genes in G4 and G7 groups increased (*p* < 0.05), while those in other groups were generally decreased (*p* < 0.05), among which G2, G3 and G6 groups were more remarkable (Figure 5E). The above results indicate that inhibition of the TGF-β signaling pathway at the early stage of endothelial differentiation repressed the mesoderm differentiation of piPSCs, while at the later stage of endothelial differentiation, TGF-β signaling inhibition promoted further endothelialization.

Finally, we detected the percentages of CD31-positive cells by flow cytometry to compare the endothelial differentiation efficiency of different groups. The results showed that the percentage of CD31-positive cells of control group was 11.87%, and the ratios of CD31-positive cells in G4 group and G7 group were higher, i.e., 14.51% and 13.84%. The ratios of CD31-positive cells of the other five groups were lower than the control group, among which G2, G3 and G6 groups were more remarkable (Figure 5F).

## 3. Discussion

Although studies have shown that transplantation of autologous vascular endothelial cells or their precursor cells improves the function of ischemic tissue and promotes angiogenesis, autologous ECs are limited and difficult to obtain [4]. As an alternative cell resource, the incomplete reprogramming and donor-specific epigenetic memory of iPSCs were once thought to impair their differentiation potential [39,40]. However, recent studies have shown that iPSC-derived ECs have good plasticity, which is of great value for understanding endothelial development and pathological changes [41].

Since transplanted vascular endothelial cells improved blood circulation in the ischemic tissues, how did they work? Some studies showed that the bone marrow-derived endothelial progenitors proliferated in the peripheral circulation and migrated to the ischemic myocardium to participate in angiogenesis [42,43]. Human iPSC-derived endothelial progenitors could integrate and repair the damaged retinal vasculature by forming new blood vessels [44]. Thus, the transplanted ECs can directly participate in angiogenesis by fusion with recipient cells or blood vessels. In addition, Cho et al., found that the number of transplanted endothelial cells continuously decreased within 1 week, but a variety of cytokines could be detected after more than 2 weeks, far beyond the secretory capacity of the cells; meanwhile, increased capillaries and decreased cardiomyocyte apoptosis could be observed [42]. Another study showed that compared with ECs directly reprogrammed from human fibroblasts, ECs derived from human iPSCs produced more cytokines, which promoted angiogenesis and tissue repair in a paracrine manner [45]. The previous studies seem to explain why in our recent experiment, despite the rapid reduction in the cells at the initial stage of transplantation, the piPSC-EC-treated group still had the best effect on tissue repair; of course, it also depends on the sensitivity of the detection method. In fact, in addition to endothelial cells, a variety of cells have been shown to function through paracrine mechanisms, e.g., the exosomes of bone marrow mesenchymal stem cells, containing growth factors, cytokines and adhesion molecules, contribute to angiogenesis [46]. Multiple myeloma cells can transfer miR135b to endothelial cells by exosomes, which directly inhibit factor-inhibitory hypoxia-inducible factor (FIH-1) and promote endothelial cell migration, proliferation and angiogenesis by the HIF-FIH signaling pathway [47].

TGF-β and its family members, emerging from an early stage of development, play crucial roles in modulating cell proliferation and differentiation, tissue specificity and organogenesis [48]. The effect of TGF-β on angiogenesis is ambiguous and even contradictory, depending on the species, stage of development and microenvironment. TGF-β played an essential role in murine yolk-sac vascular formation and maintenance; however, the addition of TGF-β inhibited the yolk-sac-like differentiation of human embryonic stem cell-derived embryoid bodies, resulting in reduced expression of endodermal, endothelial and hematopoietic markers [49,50]. Sara et al., blocked ALK1, a TGF-β type II receptor, in a mouse model of pancreatic cancer, resulting in decreased vascular density within the tumor [51]. However, silencing of the ALK1 gene in cultured ECs promoted their proliferation and migration and new blood vessel sprouting [52]. In 2013, Bai et al., found that during the derivation of vascular endothelial cells from human ESCs, the expression level of TGF-β increased from the fourth day of differentiation, and the addition of TGF-β type I receptor inhibitor SB431542 reduced the expression levels of mesodermal markers and the number of endothelial progenitors upon mesoderm induction [37]. In 2020, when Aoki et al., derived endothelial cells from human iPSCs, they found that the combination of three small molecules, namely, Y-27632 (a selective inhibitor of ROCK), A83-01 (a receptor-like kinase inhibitor of TGF-β) and CHIR-99021 (a selective inhibitor of GSK3β), improved the expansion of endothelial cells [36]. In our study, during the induction of porcine iPSCs into vascular endothelial cells, the addition of SB431542 at the early stage of differentiation inhibited the expression of mesoderm-specific genes, while the addition of SB431542 at the later stage of differentiation increased the expression of endothelial-specific genes, suggesting that TGF-β signaling plays a positive role in mesoderm formation and a negative role in its further endothelialization.

Derivation and functional studies of porcine iPSC-ECs are of great value to the clinical application of human iPSC-derived endothelial cells, which is not only reflected in the induction strategy and cell transplantation method, but also in the safety and efficacy evaluations of cell transplantation [53,54]. Although we obtained some encouraging results from the recent study, further studies are needed to clarify the paracrine mechanisms of the transplanted iPSC-ECs, as well as the safety and efficacy of autologous porcine iPSC-EC transplantation in disease pig models.

## 4. Materials and Methods

### 4.1. Differentiation of ECs from piPSCs

Differentiation of ECs from piPSCs was progressive. First, piPSCs were passaged on Matrigel (1:150) (Corning, Corning, NY, USA) to preclude the presence of mouse embryonic fibroblasts. Then, piPSCs were digested by 0.5 μM EDTA (Thermo Scientific, Waltham, MA, USA) and planted on Matrigel at a dilution of 1:10 with 10 μM ROCK inhibitor Y27632 (EMD4 Biosciences, Darmstadt, Germany). After 2 days, culture medium was switched to EC differentiation medium (FGF2 and LIF were removed from MX medium) with 6 μM CHIR99021 (Stemgent, Boston, MA, USA) for 2 days. Next, the cells were cultured in EC differentiation medium supplemented with 25 ng/mL BMP4, 10 ng/mL FGF2 and 50 ng/mL VEGF (R&D Systems, Minneapolis, MN, USA) for 2 days. At last, EC differentiation medium was changed to Endothelial Cell Growth Medium-2 BulletKit (EGM-2, Lonza, Guangzhou, China) with the addition of 50 ng/mL VEGF. The medium was changed every day [28].

### 4.2. In Vitro Tube Formation Assay

Cells were plated at a density of 1 × 10^4^ cells per well in 96-well plates coated with 50 μL growth factor-reduced Matrigel (Corning, Corning, NY, USA). Plates were incubated at 37 °C, and after 4–6 h of incubation, images were taken of each well using a Nikon 80i microscope (Nikon, Tokyo, Japan).

### 4.3. Uptake of Dil-Ac-LDL

Cells were incubated with 20 μg/mL Dil-Ac-LDL (human Dil-Acetylated Low Density Lipoprotein, YEASEN, Shanghai, China) in EGM-2 for 4 h at 37 °C. Thereafter, cells were washed three times with PBS and visualized with a Nikon 80i fluorescence microscope.

### 4.4. Immunofluorescence Staining

Cells were fixed in 4% (*w*/*v*) paraformaldehyde (PFA) at room temperature for 30 min, permeabilized in 1% (*v*/*v*) Triton X-100 in PBS at 37 °C for 1 h and blocked with 1% BSA at 37 °C for 1 h. Subsequently, the cells were incubated with primaries antibody overnight at 4 °C. After three washes with PBS, they were incubated with secondary antibodies for 1 h at 37 °C. Finally, nuclei were stained with Hoechst33342 for 5 min. Samples were visualized on Nikon 80i fluorescence microscope. Primary antibodies are listed in Table S1.

### 4.5. Flow Cytometric Analysis

On the 12th day of differentiation, adherent cells were harvested using Collagenase IV and resuspended in PBS. The cells were counted and incubated with anti-porcine CD31 antibody (porcine CD31/PECAM-1 Alexa Fluor^®^ 488-conjugated from R&D Systems, Minneapolis, MN, USA) for 30 min at room temperature, and then they were washed three times in PBS and centrifugated at 300× *g* for 5 min. After washing, the labeled cells were sorted by flow cytometry on a FACSCalibur (BD, Franklin Lakes, NJ, USA) and analyzed with Flowjo software.

### 4.6. Cell Culture on 3D Scaffold

The cells were digested into single ones and labeled with Dio cell tracker dye (Beyotime, Shanghai, China) according to the manufacturer’s instructions. Gelfoam sponges (Jinling Pharmaceutical Co., Ltd., Nanjing, China) were cut into disks with diameter of 6 mm and thickness of 2 mm using a sterile technique, and 100 μL Dio-labeled cell suspension containing 1 × 10^5^ cells was seeded onto each disk. The cells were allowed to adhere for 24 h at 37 °C, and then the gelfoam scaffolds were gently transferred into new wells of 96-well plates for further cultivation. The samples were visualized on TCS SP8 confocal laser scanning microscope (Leica, Wetzlar, Germany).

### 4.7. Mouse Ischemic Hindlimb Model and Cell Transplantation

A hindlimb ischemia model was performed with 7-week-old male NOD/SCID mice. The mice were anesthetized with avertin (1.25%) and prepared as follows: an incision of 1 cm was made near the groin and the femoral artery was ligated twice with a 5-0 silk suture. After ligation, the mice were randomly divided into 3 groups: piPSC-EC-treated group, porcine fetal fibroblasts (PFFs)-treated group and endothelial cell growth medium-2 (EGM-2)-treated group, which were injected with 1  ×  10^6^ piPSC-ECs in 200 μL of EGM-2, 1  ×  10^6^ PFFs in 200 μL of EGM-2 and 200 μL EGM-2 alone, respectively, into 3 different sites of the adductor muscle in the medial thigh. The transplanted cells were labeled with PKH26 (Sigma, Saint Louis, MO, USA), and the mice were examined on days 1, 7 and 14 using Night Owl LB983 imaging system (BERTHOLD, Bad Wildbad, Germany) [55].

### 4.8. Histological Analysis

Paraffin-embedded samples were cut into 7 μm sections using a tissue slicer (Leica, Wetzlar, Germany). Hematoxylin and eosin (H&E) staining was performed with an H&E staining kit (Beyotime, Shanghai, China). Immunohistochemistry was performed by the following steps: the sections were permeabilized with 0.1% (*v*/*v*) Triton X-100 in PBS for 5 min. After blocking with 10% (*v*/*v*) horse serum for 30 min, the sections were incubated with primary antibodies overnight at 4 °C, followed by incubation with secondary antibodies and Hoechst staining. Then, the samples were visualized by a Nikon 80i fluorescence microscope. Primary antibodies are listed in Table S1.

### 4.9. RNA Sequencing

We performed genome-wide gene expression profiling of porcine iPS cells, differentiated cells and porcine endothelial cell line AOCs via RNA deep sequencing by Annoroad Gene Technology Co., Ltd. (Beijing, China). Library construction was performed using the Illumina platform (Illumina, Inc., San Diego, CA, USA) according to the manufacturer’s instructions. The samples were sequenced on an Illumina HiSeq 2500 instrument. Three biological repeats were performed.

### 4.10. High-Throughput Data Analysis

RNA sequencing data were analyzed with the Galaxy web-based tool [56]. Principal component analysis (PCA) was conducted using R package ade4 (version 1.7-18) (https://github.com/sdray/ade4 (accessed on 21 October 2021)). Pathway analysis was performed with DAVID (gene enrichment analysis using the EASE score, a modified Fisher’s exact *p* value, as the threshold) [57]. RNA sequencing raw data were uploaded to the National Center for Biotechnology Information database under the accession number GSE200624.

### 4.11. Enzyme-Linked Immunosorbent Assay (ELISA)

The medium was collected every 24 h and centrifuged at 800 rpm/min. Into each well, 100 μL of supernatant (antigen) was added, and then the plate was sealed with a cover and incubated overnight at 4 °C. The next steps are as follows: Aspirate the antigen and wash off the unbound antigen. Add 100 μL of blocking solution into each well and incubate at 37 °C for 60 min. Aspirate the blocking solution and wash 3 times. Add 100 μL of diluted TGF-β1 antibody to each well. Seal the plate and incubate at 37 °C for 1 h. Aspirate the antibody and wash with 300 μL of washing buffer. Add 100 μL of diluted HRP conjugated secondary antibody to each well. After sealing, incubate at 37 °C for 1 h. Wash with 300 μL of washing buffer. Add 100 μL of substrate (TMB) into each well. Place the plate on a titer plate shaker and incubate for 30 min at room temperature. Add 100 μL of 0.5 M H_2_SO_4_ to stop color development. Read at 450 nm using an infinite F50 Plate Reader (TECAN, Männedorf, Switzerland) with a reference wavelength of 650 nm.

### 4.12. Western Blotting

The cells were lysed using lysis buffer RIPA (Beyotime, Nantong, China), which contains PMSF (Roche, Basel, Switzerland). Approximately 30 μg of total protein was electrophoresed on a 10% sodium dodecyl sulfate-polyacrylamide gel and electro-transferred to a PVDF membrane (Millipore, Darmstadt, Germany). The membrane was then blocked with 5% fetal bovine serum for 2 h, immunoblotted with primaries antibody and incubated with the secondary antibodies for 60 min. Primary antibodies are listed in Table S1.

### 4.13. Quantitative Real Time-PCR (qRT-PCR)

mRNA and cDNA were obtained as mentioned in the section on semi-quantitative PCR. The cDNA was used for quantitative PCR in the presence of specific primers and SYBR Green PCR Master Mix (Takara, Tokyo, Japan). Thermal cycling was conducted on a 7900HT sequence detection system (Applied Biosystems, Foster City, CA, USA). Primers for tested genes are listed in Table S2.

### 4.14. Statistical Analysis

To label piPSC-ECs and quantify their angiogenic capacity, histological sections were double-stained with anti-CD31 antibody and pig-specific anti-vimentin antibody. The capillary-like structures labeled by both antibodies were identified as the ones derived from porcine cells. Image J was used to analyze capillary density, which was presented as a percentage of capillary-like structures in a random field. For each treatment, 6–8 random fields were observed by two independent observers and then averaged. The averages were used for statistical analysis. Data were obtained from at least three independent experiments.

All statistical analyses were performed with GraphPad Prism5. A power of analysis with a 95% confidence interval was used to calculate the sample size required to obtain significant results (*p* < 0.05).

## Figures and Tables

**Table 1:** SB431542 treatment groups.

Group Name		Treatment Period	
	0–2 Day	3–4 Day	5–12 Day
Control	−	−	−
G1	+	−	−
G2	+	+	−
G3	−	+	−
G4	−	−	+
G5	−	+	+
G6	+	+	+
G7	+	−	+

+: with TGF-β inhibitor SB431542. −: without TGF-β inhibitor SB431542.

## Data Availability

The data presented in this study are available upon request from the corresponding authors.

## Supplementary Materials

The following supporting information can be downloaded at: https://www.mdpi.com/article/10.3390/ijms23137029/s1.
