# Supplementary material for: Derivation and Characterization of Endothelial Cells from Porcine Induced Pluripotent Stem Cells
Source: Int J Mol Sci. 2022 Jun 24;23(13):7029. doi: 10.3390/ijms23137029 (PMC9266935; doi:10.3390/ijms23137029)
Supplement: Supplementary file 1 [file ijms-23-07029-s001.zip › ijms-1752729-supplementary/supplementary/Supplementary materials.pdf]

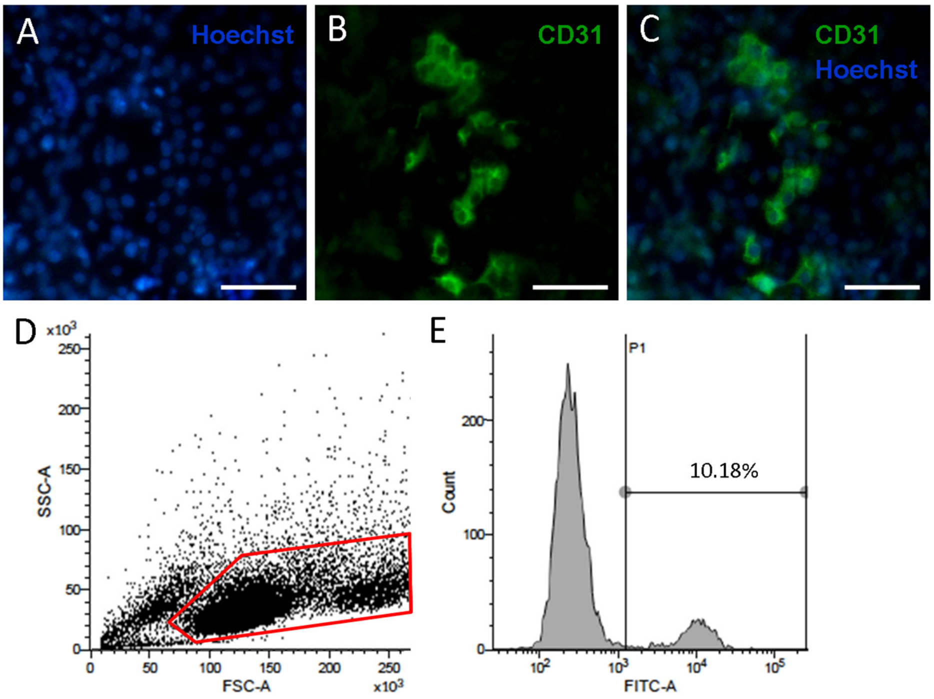

Figure S1. Immunofluorescence staining and flow cytometry sorting on day 12. (**A-C**) CD31 was detected by immunofluorescence staining. Blue fluorescence represents nuclei (**A**), green fluorescence represents CD31 positive cells (**B**), merged images of A&B (**C**). Scale bar = 50 µm. (**D**) The cells in the gate were used for analysis. (**E**) Flow cytometry sorting of CD31 positive cells on day 12 of endothelial induction.

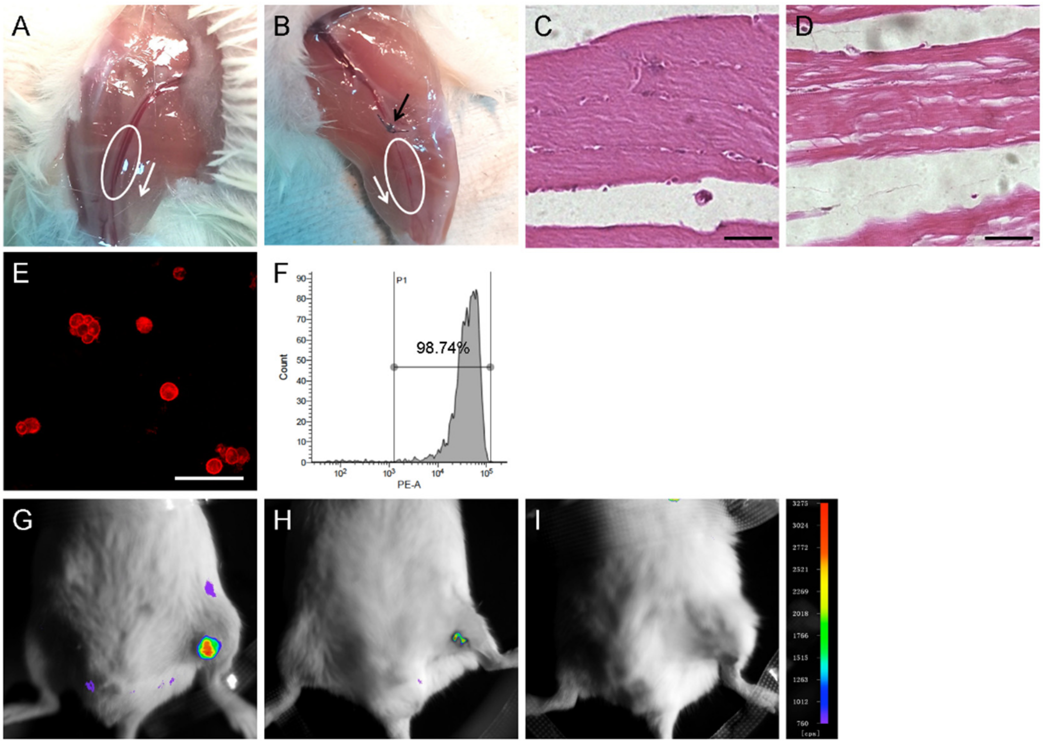

Figure S2. Establishment of mouse hind-limb ischemia model and cell transpantation(**A-B**) 7 days after ligation, anatomical observation of femoral artery in blank control group (**A**) and model group (**B**). (**C-D**) 28 days after ligation, histomorphological observation of adductor in blank control group (**C**) and model group (**D**). Scale bar=100 μm. (**E**) Porcine iPSC-ECs are labeled with PKH26. Scale bar=50μm. (**F**) Flow cytometric analysis of PKH26 positive cells. (**G-I**) Fluorescence imaging of porcine iPS-ECs in the ischemic hind limb of mice after 1 day (**G**), 7 days (**H**) or 14 days (**I**) of cell transpantation.

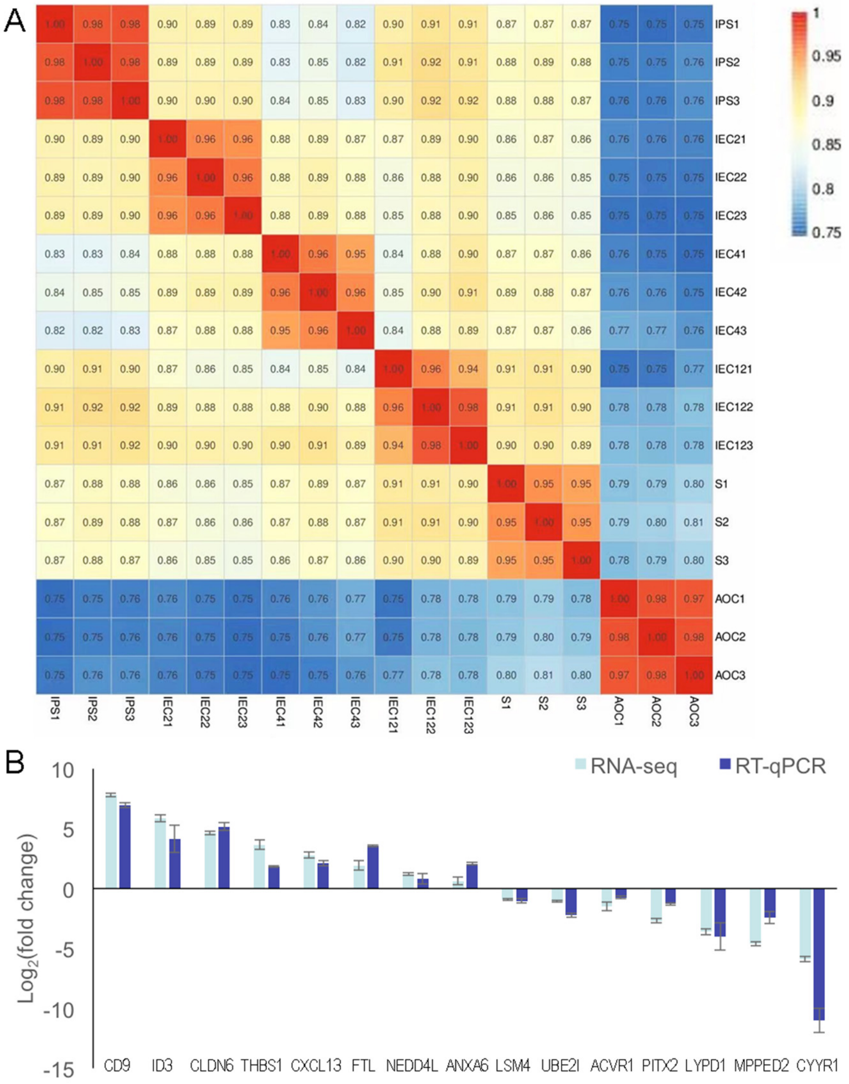

Figure S3. Correlation analysis and RT-qPCR verification. (**A**) Correlation analysis of genome-wide transcriptional profiles among samples. (**B**) RT-qPCR was used to verify the reliability of RNA-sequencing data.

Table S1. Information of primary antibodies

Protein	Catalogue	Supplier
VE-cadherin	sc-9989	Santa cruz biotechnology
vWF	ab6994	Abcam
eNOS	ab5589	Abcam
Porcine CD31Alexa Fluor® 488	FAB33871G	R&D Systems
CD31	ab28364	Abcam
Vimentin	V6630	Sigma
SMAD2	GTX111075	Gene Tex
pSMAD2	GTX133614	Gene Tex
SMAD3	ab40854	Abcam
pSMAD3	Ab118825	Abcam
TGF-β1	PA1-9574	invitrogen
β-Actin	A1978	sigma

Table S2. Information of quantitative-PCR primers

Gene name	Primer sequence (5′→3′)	Primer sequence (3′→5′)	Length （bp)
GAPDH	GCAAAGTGGACATTGTCGCCATCA	TCCTGGAAGATGGTGATGGCTT	160
brachyury	GCCAGATCATGCTGAACTCCTTA	ATAAGCCGTCACCGCTATGAAC	128
MIXL1	GTCTTCCGCAGGACCATGTA	TCAAATATCTCCCTTCCGTTTC	205
PDGFR-2	GGTCACCTGTGCCGTCTTTA	TTTGATGGACGGGACCTTGG	115
Mesp1	CGTCTTGGGGGTCTCCTTCTG	GGGGCCAATATTCCACCGTC	225
Mesp2	CAACGCAGCCCTTTGGATG	GGGTTTCTGGTGACAGATAGGACTC	167
CD31	CGAGGTCTGGGAACAAAGGG	AGCCTTCCGTTCTAGAATATCTGTT	97
VE-cadherin	CCAAGCCCTATGAGGCCAAA	AGTTGCTGTCCTCAGTGCTC	135
VEGFR-2	GAGCCCCTGATTACACCACC	GCAGATACTGACTGATTCCTGCT	297
CD9	TTCATCTTCTGGCTCGCTGG	CGCCGGCTCCAATGAGAATA	142
ID3	CTCGCTCCGGAACTTGTGAT	AGCACCTGCGTTCTGGAAG	86
CLDN 6	ATATGGCCCGATACTCGGCA	CACCCCCACCCGAATTAGAC	94
THBS1	CAGCATCCGCAAAGTGACTG	CTGGACTCCGTTGTGGTAGC	85
CXCL13	TGTCCAACCAGAGAAGTCATAGTC	ACACTGGAGCTGGTAAAGTTGT	136
FTL	CATGAGCTCCCAGGTTCGTC	GCGGTTGAAATAGAAGCCCAGA	121
NEDD4L	CTTCGGAGCCAGTGATCCAT	TCCACTTTGGGTTCAGCGTC	113
ANXA6	TGCTGGCTGTAGTGAAGTGT	GAATCAGGGTGTTGTCCCGA	102
LSM4	CGTTGCTGCCACTCCATTTC	CGACAAGGGAAGCATGGTGT	127
UBE2I	CGTTATTCCACCCGAACGTG	TCCTGTATTCCTAACAAGATCTGC	115
ACVR1	TGCATAGCAGATTTGGGCCT	GAACTTCGGGGGCCATGTAA	109
PITX2	CGCTCCCTGGACTCTTCCAA	GGCTCTTGTCTTTCTCTGCGG	88
LYPD1	TCTTGCTTCAAGGGTTGGCG	GCAATTCACGATGAACTCGGG	98
MPPED2	AGCCCGGGAAAGAAACACG	TGTGCCATCCTTCCTCCCTA	141
CYYR1	GGTGCTGCTCTTTGTCTACG	TGGCTGTGCCCGAGAGA	137

Table S3. Genes differently expressed between piPSCs and AOCs

(Excel table)
